# Proactive personality and critical thinking in Chinese medical students: The moderating effects of psychological safety and academic self-efficacy

**DOI:** 10.3389/fpsyg.2022.1003536

**Published:** 2022-10-17

**Authors:** Yan-ping Wang, Chen-xi Zhao, Shu-e Zhang, Qing-lin Li, Jing Tian, Mao-ling Yang, Hai-chen Guo, Jia Yuan, Sheng-yan Zhou, Min Wang, De-pin Cao

**Affiliations:** ^1^Department of Health Management, School of Health Management, Harbin Medical University, Harbin, China; ^2^Academic Affairs Office, First Affiliated Hospital of Harbin Medical University, Harbin, China; ^3^Department of Obstetrics, Women and Children’s Hospital Affiliated to Chengdu Medical College, Chengdu, China

**Keywords:** critical thinking, proactive personality, psychological safety, academic self-efficacy, medical students

## Abstract

**Objectives:**

This study aimed to identify the relationship among proactive personality, psychological safety, academic self-efficacy and critical thinking, and to further explore whether psychological safety and academic self-efficacy could be a moderator in the association between proactive personality and critical thinking among Chinese medical students.

**Materials and methods:**

The cross-sectional study was carried out from October to December 2020 in China. Totally, 5,920 valid responses were collected at four Chinese medical universities. Critical thinking, proactive personality, psychological safety, academic self-efficacy and demographic factors were assessed through questionnaires. Hierarchical multiple regression was used to identify interrelationship clusters among variables. Simple slope analyses were performed to explore the moderating effects of psychological safety and academic self-efficacy.

**Results:**

The mean score of critical thinking among medical students was 3.85 ± 0.61. Proactive personality, psychological safety, and academic self-efficacy were shown to be important factors for critical thinking. Psychological safety and academic self-efficacy moderated the association between proactive personality and critical thinking. A simple slope analysis showed that high psychological safety and academic self-efficacy weakened the impact of proactive personality on critical thinking.

**Conclusion:**

Most medical students surveyed in China might have relatively high levels of critical thinking. Psychological safety and academic self-efficacy moderated the association between proactive personality and critical thinking. More interventions related to psychological safety and academic self-efficacy will be helpful to improve critical thinking among Chinese medical students.

## Introduction

Critical thinking, regarded as a kind of individual rational thinking of an introspective nature, consists of the synthesis of knowledge, attitudes, and skills (Ennis, 1989). It is divided into two major characteristics: introspection and questioning (Ennis, 1989). The American Philosophical Association emphasizes that critical thinking is a comprehensive capacity that includes purposeful self-adjustment, the active and skillful gathering of information, and summarizing, applying, and analyzing this information (Facione et al., 1994). Generally, critical thinking is considered a key training outcome in the health sciences programs in higher education (Styers et al., 2018). The “Global Minimum Essential Requirements in Medical Education,” published by the Institute for International Medical Education, emphasizes the significance of critical thinking ability for medical students with undergraduate degrees (Schwarz and Wojtczak, 2002). In addition, the Standards for Basic Medical Education in China (for trial implementation) formulated by the Ministry of Education’s Working Committee for the Accreditation of Medical Education, also highlights the importance of a series of criteria for medical graduates, including scientific attitude, innovation spirit, and analysis and critical spirit (Ministry of Education of the People’s Republic of China, 2008). A large amount of literature suggests that critical thinking ability enables medical graduates to better understand complex patient situations in future medical practice (Zori et al., 2010; Pitt et al., 2015). In addition, when medical students with critical thinking ability are faced with conflicts in clinical practice between old and new knowledge, they can compare the knowledge before taking action (Holmes et al., 2015). They can also break through existing knowledge and make use of old and new knowledge to innovate medical science (Eggers et al., 2017). Cultivating medical students’ critical thinking ability is of great significance in improving their adaptability to future work and in promoting the development of medical science. Due to the differences between Chinese and western cultures, there was difference in critical thinking Chinese and Western students. Therefore, considering practical value in medical education, the current research specifically focused on critical thinking’ influence mechanism among medical students based on the context of Chinese Oriental culture.

Critical thinking is not only restricted by the external environment, but it is also regulated by personality in the process of cultivation (Friedman, 2004). To date, research has found that a proactive personality is the most predictive personality trait for job performance (Thompson, 2005). Proactive personality is a relatively stable behavioral tendency and personality characteristic. It primarily refers to the behavioral tendency of individuals to constantly explore new paths, seize new opportunities, and take action that can change the external environment without being restricted by external resistance (Bateman and Crant, 1993). Based on self-determination theory, medical students with proactive personalities, who are influenced by internal and external motivations, will choose to self-reflect and ask questions to gain more initiative (Sheldon and Krieger, 2007). Therefore, we inferred that proactive personality could be protective factor for critical thinking among medical students as mentioned above and the existed evidence (Tu and Lin, 2018). Moreover, the specific and detail mechanism of the relationship between proactive personality and critical thinking were still unclear. For instance, what psychological conditions may reinforce the relationship between proactive personality and critical thinking. Previous studies demonstrated that college students are more likely to suffering from psychological problems, especially medical student (Yusoff et al., 2013; Bacchi and Licinio, 2015). Medical students are in a passive receiver and negative thinker under Chinese didactic lecture teaching pattern, which may lead to psychological problems and further affect critical thinking (Chen et al., 2010; Deng et al., 2014). It is necessary for scholars that further research focus on the deepen understanding of the psychological conditions mechanisms between proactive personality and critical thinking for medical students under Chinese educational context. Therefore, this study attempts to explore some psychological mechanisms in the relationship between medical students’ proactive personality and critical thinking from the perspective of educational promotion. It is worth considering how psychological factors influence the relationship between proactive personality and critical thinking, and how to use the possible conclusion to improve medical students’ critical thinking ability.

Psychological safety, as a determinant of mental health, has been widely studied (Wang et al., 2019). The original concept of psychological safety was defined by Edmondson through his research on a model of team learning, he pointed out that psychological safety was a specific confidence, belief and feeling among team members. It is an individual feeling of potential interpersonal risks in their surrounding environment (Edmondson, 1999). About the psychological safety of students in schools, Jeroen defined psychological safety as the feeling that students were able to show and employ themselves in their tasks without fear of negative consequences to self-image, social status, or school career (Schepers et al., 2008). It is to say that psychological safety is a subjective judgment of the certainty and controllability of the environment, which is a state of consciousness based on personal characteristics. Therefore, we agreed with Schepers that psychological safety is regarded as a kind of psychological state that makes students feel safe, comfortable, relaxed, and stable.

However, existing research into psychological safety mostly focuses on the workplace (Carmeli et al., 2009; Hu et al., 2018). Previous studies have suggested that a safe psychological status eliminates fear and tension about negative outcomes among employees, leading to improved positive personality and knowledge sharing as well as learning behaviors (Gong et al., 2012; Frazier et al., 2017). Moreover, previous research into the relationship between personality and security also indicates that personality can predict security among Chinese adolescents (Pan et al., 2018). Medical students influenced by their own personality characteristics often have different levels of psychological safety, which affects their criticism and innovation behavior (De Dreu and West, 2001). Furthermore, psychological safety is often explored as a mediating variable in a series of relationships (Jia et al., 2017). However, few studies have explored the moderating mechanism among medical students in Chinese cultural background, which is different from the western culture. Supplementally, we regarded psychological safety as a moderator in the relationship between proactive personality and critical thinking among Chinese medical students.

Self-efficacy is a research area in positive psychology (Ouweneel et al., 2013). The theoretical framework of Bandura’s social cognitive theory proposes that self-efficacy is a structure that reflects a person’s confidence in their ability to successfully perform an action (Bandura, 1977, 2001). Academic self-efficacy is the academic performance of self-efficacy, which refers to the belief and motivation that students can achieve the expected academic level (Zimmerman, 1995). Bandura (1993) proposed four sources of self-efficacy: past performance, vicarious experience, verbal persuasion, and physiological/emotional state. These sources indirectly influence individuals’ behavior patterns (e.g., choice and persistence) and thought patterns (e.g., goals and attributions) through their influence on efficacy expectations (Bandura, 1993). Regarding academic performance, medical students with proactive personalities are more active in their studies and have great enthusiasm and confidence in completing their learning tasks (Wu et al., 2020; Chen et al., 2021). Moreover, to obtain more knowledge, better grades, and psychological satisfaction, they constantly question and reflect on the learning process and results (Nur’azizah et al., 2021). Academic self-efficacy is often used as a moderating factor in previous study (Liu et al., 2022). Existing studies have confirmed the relationship between academic self-efficacy and critical thinking (Dong, 2016; Kim and Kim, 2016). Studies have also confirmed the correlation between proactive personality and academic self-efficacy (Lin et al., 2014; Chen et al., 2021). However, the role of academic self-efficacy in the relationship between proactive personality and critical thinking remains unclear. Whether academic self-efficacy has a moderating effect on proactive personality’s impact on critical thinking is worthy of further investigation.

In summary, the present study aims to evaluate the critical thinking level of Chinese medical students and explore the relationship between proactive personality, psychological safety, academic self-efficacy, and critical thinking. And to further examine whether psychological safety and academic self-efficacy has a moderating effect in the relationship between proactive personality and critical thinking.

## Materials and methods

### Participants and procedures

Considering timeliness, cost effectiveness, and accessibility, this study conducted a cross-sectional anonymous online survey in China’s Heilongjiang Province from October 2020 to December 2020. A convenience sampling method was used to collect data from the medical students. Students from Harbin Medical University (including Daqing Campus), Jiamusi University, Qiqihar Medical University, and Mudanjiang Medical University were selected as the research subjects. According to Zhou et al.’s (2017) calculation method and standard requirements for the cross-sectional sample size, the minimum sample size of this study was calculated to be 1,824 participants. Given the actual response rate of only 50%, and the problem of questionnaire quality control, a preliminary survey of 3,648 participants was finally conducted.

The specific procedures were as follows. First, the research was conducted following the guidelines of the Declaration of Helsinki and approved by the ethics committee of the Institutional Review Board at Harbin Medical University. All subjects provided informed consent to participate in this study. All information obtained was anonymous and confidential to protect the privacy of the study subjects. Second, before the investigation phase of the study, the researcher sent the research specifications to the educational administrators at the target university and obtained their consent and cooperation. The purpose and significance of the research were explained by the school administrator to the department counselors, who then handed it out to the students in each class to fill out voluntarily. Third, the survey was distributed through the online research platform “Questionnaire Star.” The questionnaire was sent to medical students *via* a mobile phone link, and the questionnaire was completed only once per Internet Protocol address. Researchers used the Questionnaire Star platform to conduct real-time monitoring and collate the survey data. Another investigator controlled the quality of the collected questionnaires. Those with short answer times, fixed answering modes, and conflicting answers to reverse questions were excluded. Finally, we collected 5,920 valid questionnaires.

### Measurement of critical thinking

Critical thinking was measured using Jiang’s five-item revised version of the 10-item California Critical Thinking Disposition Inventory (Jiang and Yang, 2014; Snell and Lefstein, 2018). The results were graded on a five-point Likert scale (ranging from 1 = very much disagree to 5 = very much agree). The total score ranged from 5 to 25. The higher the score, the higher the individual’s critical thinking tendency. In the current study, Cronbach’s α for the total scale was 0.890.

### Measurement of proactive personality

Proactive personality was measured using a simplified version of the Proactive Personality Scale (Parker and Sprigg, 1999), which has been used in many studies to measure the level of proactive personality and includes six items (Wu and Ma, 2019). The results were graded on a five-point Likert scale (ranging from 1 = very much disagree to 5 = very much agree); the higher the score, the stronger the individual’s proactive personality tendency. Cronbach’s α for the total scale was 0.826.

### Measurement of psychological safety

Psychological safety was measured using the Psychological Safety Scale (Edmondson, 1999; Wang et al., 2019), which has seven items. Li et al. pointed out that Edmondson’s scale was originally used to measure psychological safety at the team level (Li and Tan, 2013). In order to measure psychological safety at the individual level, they selected items unrelated to other team members, and this method has good reliability (Li and Tan, 2013). In current study, we measured seven-item psychological safety scale at the individual level modified (Li and Tan, 2013). Examples of scale items include “Even if I make mistakes in class, I will not be criticized by my teachers or classmates.” “In our class, students are allowed to take risks (challenge the teacher, ask bold questions, etc.)” Combined with the content from qualitative interviews, the context of the items was modified to conform to the situation of this study. The results were graded on a seven-point Likert scale (ranging from 1 = very much disagree to 7 = very much agree); the higher the score, the higher the degree of perceived psychological safety. Cronbach’s α for the psychological safety scale was 0.872.

### Measurement of academic self-efficacy

Academic self-efficacy was measured using an eight-item scale modified from the self-efficacy part of the “Learning Motivation Strategy Questionnaire” developed (Garcia and Pintrich, 1996; Wang and Lin, 2007). The results were graded on a seven-point Likert scale (ranging from 1 = very much disagree to 7 = very much agree); the higher the score, the higher the degree of academic self-efficacy. Cronbach’s α for the learning self-efficacy scale was 0.956.

### Measurement of medical students’ demographic characteristics

Eleven demographic characteristics were collected for this study, including gender, grade, educational system, major, origin of student, one-child, first-generation college student, parenting style, academic performance, experience of leadership cadre, and inclined classroom seats. Parenting style was divided into authority type (strict requirements and more companionship), authoritarian type (strict requirements and less companionship), tolerant type (less strict requirements and more companionship), and neglected type (less strict requirements and less companionship) (Maccoby and Martin, 1983). The experience of leadership cadre refers to whether the student is in a class cadre or has joined an autonomous management organization such as the student union. Responses were divided into “yes” and “no.” Academic performance was divided into four grades: top 25, 26–50%, 51–75%, and bottom 25%. Inclined classroom seats were divided into front, middle, and back rows.

### Statistical analyses

All statistical analyses were performed using IBM SPSS Statistics 21.0, with a two-tailed value of *p* < 0.05 considered to be statistically significant. The correlation of continuous variables was detected using Pearson correlation analysis. A series of hierarchical multiple regressions were applied to examine the association between proactive personality, psychological safety, academic self-efficacy, and critical thinking. The moderating effect of psychological safety and academic self-efficacy on the relationship between proactive personality and critical thinking was explored by adding interaction items (Wen et al., 2005). If the interaction effect was statistically significant, a simple slope analysis was conducted to visualize the interaction term. In the simple slope analysis, for continuous moderators the value at the mean of *z* and at 1 *SD* above and below the mean of *z* were selected as the cut-off points for high and low levels based on the suggestion of scholars (Cohen et al., 1983). The multicollinearity among all variables was evaluated by variance inflation factor (*VIF*); *VIF* < 10 was considered acceptable (Song et al., 2019). In the present study, no problematic *VIF* (> 10) was identified in any model.

## Results

### Demographic characteristics of participants

A total of 5,920 medical students answered the questionnaire, 28.6% of which were male and 71.4% were female. Half of the respondents were five-year students, accounting for 48.2%, as presented in Table 1.

**Table 1:** Demographic characteristics of study participants (*N* = 5,920).

**Characteristics**	*N* (%)	
	*N*	%
**Gender**		
Male	1,691	28.56
Female	4,229	71.44
**Grade**		
Freshman	2,363	39.92
Sophomore	1,265	21.36
Junior	1,506	25.44
Senior	647	10.93
Senior 5 and above	139	2.35
**Educational system**		
Three year system	279	4.71
Four year system	2,471	41.74
Five year system	2,853	48.19
“5 + 2” or seven-year system	106	1.79
“5 + 3” year system	211	3.57
**Major**		
Medical	3,183	53.77
Medical technology	844	14.26
Pharmacy	921	15.56
Nursing	388	6.55
Biological Sciences	25	0.42
Others	559	9.44
**Origin of student**		
City	2,527	42.69
Countryside	3,393	57.31
**The one-child**		
Yes	2,852	48.18
No	3,068	51.82
**First-generation college student**		
Yes	3,922	66.25
No	1998	33.75
**Parenting style**		
Neglected type	623	10.52
Tolerant type	3,665	61.91
Authoritarian type	652	11.01
Authority type	980	16.56
**Academic performance ranking**		
Top 15%	2,614	44.16
26–50%	1738	29.36
51–75%	1,077	18.19
Last 25%	491	8.29
**Experience of leadership cadre**		
Yes	2,923	49.38
No	2,997	50.62
**Inclined classroom seats**		
Front row	2,287	38.63
Middle row	3,118	52.67
Back row	515	8.70

### Correlations among continuous variables

Table 2 showed the correlation between proactive personality, psychological safety, academic self-efficacy, and critical thinking. As shown in Table 2, critical thinking was positively correlated with proactive personality (*r* = 0.751, *p* < 0.01), psychological safety (*r* = 0.514, *p* < 0.01), and academic self-efficacy (*r* = 0.634, *p* < 0.01). In addition, proactive personality was also positively correlated with psychological safety (*r* = 0.477, *p* < 0.01) and academic self-efficacy (*r* = 0.594, *p* < 0.01), while psychological safety was still positively correlated with academic self-efficacy (*r* = 0.600, *p* < 0.01).

**Table 2:** Correlation coefficients among continuous variables of medical students (*N* = 5,920).

Variables	*Mean ± SD*	1	2	3	4
1 Proactive personality	3.68 ± 0.58	1			
2 Psychological safety	4.96 ± 0.95	0.477^**^	1		
3 Academic self-efficacy	5.10 ± 1.04	0.594^**^	0.600^**^	1	
4 Critical thinking	3.85 ± 0.61	0.751^**^	0.514^**^	0.634^**^	1

^**^p < 0.01 (two-tailed).

### Hierarchical regression analyses

**Figure 1:**
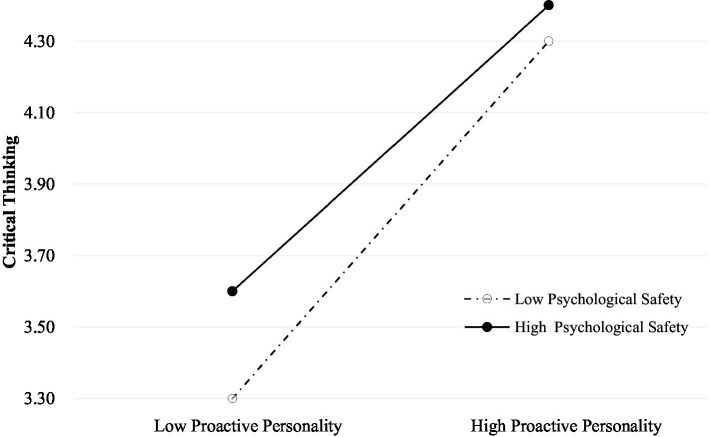
Simple slope plot of the interaction between proactive personality and psychological safety on critical thinking.

Table 3 showed the results of the hierarchical regression analyses. In Model 1, the demographic variables were input as control variables. These included gender, grade, educational system, major, origin of student, one-child, first-generation college student, parenting style, academic performance ranking, experience of leadership cadre, and inclined classroom seats. Model 2 added proactive personality and psychological safety to the list of variables used for Model 1. We found that proactive personality was positively correlated with critical thinking (*β* = 0.647, *p* < 0.01), and psychological safety was positively correlated with critical thinking (*β* = 0.202, *p* < 0.01). The addition of proactive personality and psychological safety improved the degree of fit of the critical thinking model (*adjusted R*^2^ = 0.598, *∆R*^2^ = 0.558, *p* < 0.01). Model 3 added the interaction term between proactive personality and psychological safety to the list used for Model 2. The results of the study indicated that proactive personality × psychological safety interaction items were significantly and negatively associated with critical thinking (*β =* −0.160, *p* < 0.01). Psychological safety plays a moderating role in the relationship between proactive personality and critical thinking. Simple slope analysis revealed that when psychological safety is higher, the association between proactive personality and critical thinking becomes weaker. In other words, the slope of low psychological safety was higher than that of high psychological safety, and the slope of low psychological safety was more inclined. Compared with low psychological safety, high psychological safety weakened the influence of proactive personality on critical thinking. The interaction is visualized in Figure 1.

**Table 3:** Hierarchical multiple regression results of critical thinking among medical students (*N* = 5,920).

**Variables**	**Critical thinking**				
	M1*(β)*	M2*(β)*	M3*(β)*	M4*(β)*	M5*(β)*
**Control variables**					
Gender	−0.038^**^	0.027^**^	0.025^**^	0.025^**^	0.023^**^
Grade	−0.019	−0.022^**^	−0.023^**^	−0.028^**^	−0.028^**^
Educational system	0.000	−0.006	−0.007	−0.004	−0.004
Major	−0.012	−0.013	−0.012	−0.004	−0.004
Origin of student	−0.033^*^	0.002	0.002	0.003	0.003
The one-child	−0.034^*^	−0.012	−0.012	−0.014	−0.015
First-generation college student	0.001	−0.005	−0.005	−0.003	−0.003
Parenting style	0.038^**^	0.016	0.016	0.008	0.008
Academic performance ranking	−0.103^**^	−0.031^**^	−0.030^**^	0.010	0.011
Experience of leadership cadre	−0.090^**^	−0.017^*^	−0.018^*^	−0.010	−0.010
Inclined classroom seats	−0.073^**^	−0.013	−0.013	0.008	0.008
**Predictor variable**					
Proactive personality		0.647^**^	0.800^**^	0.576^**^	0.681^**^
**Moderator variable**					
Psychological safety		0.202^**^	0.206^**^		
Academic self-efficacy				0.295^**^	0.294^**^
Proactive personality × psychological safety			−0.160^**^		
Proactive personality × academic self-efficacy					−0.109^**^
*F*	23.241^**^	678.851^**^	634.596^**^	741.637^**^	691.035^**^
Adjusted *R*^2^^**^	0.040	0.598	0.600	0.619	0.620
*∆R* ^2^^**^	0.041	0.558	0.601	0.579	0.621

^*^p < 0.05; ^**^p < 0.01 (two-tailed); *β* is the normalized regression coefficient.

**Figure 2:**
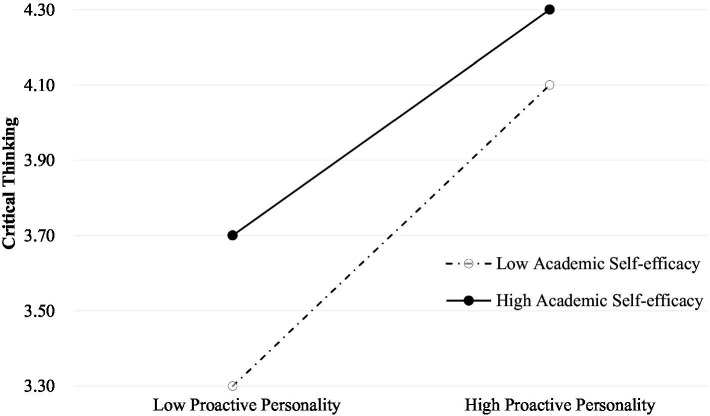
Simple slope plot of the interaction between proactive personality and academic self-efficacy on critical thinking.

Another regression analysis was conducted repeating the Model 1. Model 4 added proactive personality and academic self-efficacy to the list of variables used for Model 1. The results showed that proactive personality (*β* = 0.576, *p* < 0.01) and academic self-efficacy (*β* = 0.295, *p* < 0.01) were positively correlated with critical thinking. Moreover, we included the interaction item of proactive personality × academic self-efficacy, shown in the table as Model 5. The results showed that the interaction term between proactive personality × academic self-efficacy was negatively associated with critical thinking (*β* = −0.109, *p* < 0.01). Academic self-efficacy played a moderating role in the relationship between proactive personality and critical thinking. As shown in Figure 2, high academic self-efficacy relative to low academic self-efficacy weakens the influence of proactive personality on critical thinking.

## Discussion

### Current situation and analysis of medical students’ critical thinking

The results of this study show that the average critical thinking score of the Chinese medical students surveyed was 3.85 ± 0.61. The critical thinking score was slightly higher than the theoretical median of 3, which is above the average level (Liu, 2017). This indicates that Chinese medical students show a relatively higher average tendency toward critical thinking, which is consistent with the findings of other scholars (Yeh, 2002; Huang et al., 2018). However, some scholars have found that the tendency for critical thinking among medical students in China is relatively lower than in Western countries, such as the United States (Colucciello, 1997; Tiwari et al., 2003). This may be due to the impact of China’s medical education personnel training goals and cultural differences between the East and West. Power distance theory may provide a perspective for understanding the current results, particularly difference in Eastern and Western countries (Wanda et al., 2014). Power distance is the degree to which societies expect and accept an unequal distribution of power (Minnis, 1999), and it is particularly pronounced in hierarchical societies (Kim and Cha, 2013). There are considerable differences in power distance under Chinese culture and Western culture. Hofstede believed that China is a high-power distance culture, compared to the Western countries (Hofstede, 1991). In Chinese culture with high-power distance, people take for granted the existence of power hierarchies in society (Fu, 2008). Differences in power distance main caused by culture difference. Compared to Western Christian culture (Page and Wiseman, 1993), China with the Confucian culture is characterized by the Confucian ethics of differential love and the idea of maintaining a hierarchy (Page and Wiseman, 1993; Scarborough, 1998). Chinese Confucianism has had a profound influence on the Chinese people (Scarborough, 1998), including students. Especially, the power distance is relatively larger between teachers and students in medical university setting. Teachers are perceived to hold higher level positions, thus they are held in high esteem and students are less likely to challenge what is taught (Kim and Cha, 2013). Therefore, medical students tend to form teacher-dominated interaction style in the teaching interaction between teacher and student, which may hinder the cultivation of students’ critical thinking ability (Kawashima and Petrini, 2004). Under medical educational context, medical teachers are perceived to hold higher level positions, reputation and power and believe that it is their responsibility to ensure students learning that they taught, thus they are held in high-level academic authority and students are less likely to challenge what is taught (Stockhausen, 2007; Chiang et al., 2010). In addition, Chinese people’s traditional utilitarian motivation hinders the development of critical thinking (Yan, 1998). Medical students study for the purpose of examination performance, ignoring their own interesting and initiative, which limits, to some extent, the cultivation of critical thinking (Cox, 2009). Meanwhile, traditional medical education focuses on improving students’ professional knowledge and skills (Foster and Lemus, 2015), paying less attention to students’ thinking ability in developing medical talent (Knowles and Gray, 2011; Foster and Lemus, 2015). Therefore, a relevant suggestion is to identify the key factors and intervention measures of critical thinking to cultivate medical students’ critical thinking, such as assessment and evaluation on critical thinking ability.

### The association between proactive personality and critical thinking

The results verified that the medical students’ proactive personality is correlated with critical thinking. These results are consistent with those reported in other researcher and is exactly as expected. In research on the relationship between personality, cognition, and behavioral style (Curry, 1983), critical thinking ability belongs to the tendency toward information processing at the cognitive level, which is not only restricted by the external environment but also regulated by internal personality (Curry, 1983). This study also confirmed that psychological safety and academic self-efficacy play a moderating role in the relationship between proactive personality and critical thinking.

#### Proactive personality has a greater impact on critical thinking with lower psychological safety

The simple slope analysis indicated that the slope of low psychological safety was significantly higher than that of high psychological safety. Thus, the proactive personality of medical students with low psychological safety has greater influence on critical thinking. This study attempted to interpret this result from the perspective of cultural differences between Eastern and Western countries. Unlike Western culture, Eastern culture is based on social orientation rather than individual standards (Luomala et al., 2015). Since ancient times, Westerners have liked to take risks and explore, while the people in China have placed more emphasis on stability and security instead of adventure and exploration (Luomala et al., 2015). This is because, in China, medical students with low psychological safety find it easy to break through their own psychological defense line and have the courage to pursue changes in themselves and the external environment (Thau et al., 2009). However, those with high psychological safety pursue more stability and are not prone to active change (Thau et al., 2009). Therefore, such a tendency may interfere with the effect of proactive personality and reduce its influence on critical thinking. The present results suggest that interventions for medical students with low psychological safety is beneficial to better cultivate their critical thinking ability in China. A safe and attractive learning environment should be built within Chinese medical education to improve the psychological safety of medical students, thus promoting their critical thinking (Roh et al., 2020). In addition, peer mentoring might be an effective solution for building psychological safety (Dokal et al., 2020). Hence, teachers and medical students should actively build good peer relationships.

#### Proactive personality has a greater impact on critical thinking with lower academic self-efficacy

The simple slope analysis indicated that high academic self-efficacy weakens the impact of proactive personality on critical thinking. According to the self-determination theory, medical students with proactive personality, under the action of internal and external motivation, they will choose to reflect and question to obtain more initiative (Sheldon and Krieger, 2007). The process of producing critical thinking can also be regarded as an individual initiative (Bandura, 1977, 2001). Academic self-efficacy is the product of social cognition, a comprehensive understanding and effective evaluation of the individual, and the basis of individual initiative. For medical students with high academic self-efficacy, positive psychological state (such as positive emotions, active knowledge sharing, etc.) would be more conducive to the generation of individual proactive behaviors. Considering critical thinking is typical active behavior, medical students with high academic self-efficacy will constantly reflect on and improve their behavior in the process of learning because they have great enthusiasm and confidence to complete their studies. On the contrary, when medical students have low academic self-efficacy, their negative psychological state will not be conducive to the generation of individual initiative behavior. At this time, the active personality factors of medical students will have greater influence on critical thinking. According to the simple slope plot, compared with students with high academic self-efficacy, students with low academic self-efficacy have a stronger positive relationship between proactive personality and critical thinking. As shown in Figure 2, the slope of high academic self-efficacy is smaller, and the effect of high academic self-efficacy still exists, and its growth rate is slower. The slope of low academic self-efficacy is larger and more skewed, increasing at a faster rate. Therefore, interventions for medical students with low academic self-efficacy should have a more visible intervention effect on critical thinking (Chen et al., 2021). This study suggests that teaching strategies can be appropriately adjusted to stimulate medical students’ enthusiasm for learning and improve their learning interest in the process of teaching, such as problem-based learning, flipped classrooms, and other teaching modes (Khoiriyah et al., 2015; O’Flaherty and Costabile, 2020). Moreover, teachers should pay considerable attention to improving students’ learning confidence through encouragement and support (Tsuei et al., 2019; Zhen et al., 2020). Teachers should guide medical students to learn appropriate self-attribution and establish appropriate learning objectives to improve their sense of academic self-efficacy and further cultivate critical thinking (Perry and Perry, 1983).

### Limitations

Although this study has produced some valuable findings regarding the moderating effects of proactive personality on critical thinking, some limitations still must be addressed. First, a stratified multi-stage sampling method was used to collect data from four regions in China, which may limit the generalizability of this study to other regions. Second, as a cross-sectional study, only correlation, rather than causality, can be obtained. Third, although a series of quality control measures were adopted, there may have been uncertain deviations in the online data collection. Therefore, more rigorous sampling techniques and larger sample sizes from different cultural regions are needed in the future.

## Conclusion

In summary, this study found the critical thinking tendency of Chinese medical students to be above average. Moreover, our findings indicated that proactive personality, psychological safety and academic self-efficacy were protective factors for critical thinking. Psychological safety and academic self-efficacy play moderating roles between proactive personality and critical thinking. Furthermore, relatively high psychological safety and relatively high academic self-efficacy weaken the influence of proactive personality on critical thinking. These findings suggest that policymakers and managers in medical universities should pay close attention to the psychological factors of Chinese medical students. It emphasizes the importance of intervening these psychological factors to improve the critical thinking of Chinese medical students. Such as, educators need to incorporate formative evaluation involved critical thinking ability into performance evaluation to make up for the shortcomings of final assessment. Potential interventions might include (but are not limited to) strengthening psychological safety and academic self-efficacy to improve critical thinking among Chinese medical students.

## Data availability statement

The raw data supporting the conclusions of this article will be made available by the authors, without undue reservation.

## Author contributions

Y-pW, C-xZ, and S-eZ came up with the idea and designed the study with the help of D-pC. Acquisition of data was done by Q-lL and JT with help from M-lY and H-cG. JY and S-yZ entered the data into SPSS with the help from MW. Y-pW analyzed and interpreted the data with the help from C-xZ. Y-pW, S-eZ, and D-pC conducted the focus group discussion. All authors contributed to the article and approved the submitted version.

## Conflict of interest

The authors declare that the research was conducted in the absence of any commercial or financial relationships that could be construed as a potential conflict of interest.

## Publisher’s note

All claims expressed in this article are solely those of the authors and do not necessarily represent those of their affiliated organizations, or those of the publisher, the editors and the reviewers. Any product that may be evaluated in this article, or claim that may be made by its manufacturer, is not guaranteed or endorsed by the publisher.
